# Forestier Disease as a Cause of Dysphagia

**DOI:** 10.31138/mjr.140923.fdd

**Published:** 2023-09-14

**Authors:** Catarina Dantas Soares, Nuno Madureira, Daniela Santos-Faria

**Affiliations:** 1 Rheumatology Department, Local Health Unit of Alto Minho, Hospital Conde de Bertiandos, Ponte de Lima, Portugal,; 2 Centre for Rehabilitation Medicine in the Centro-Rovisco Pais Region, Portugal

**Keywords:** dysphagia, hyperostosis, Forestier Disease, metabolic disorder

## INTRODUCTION

Diffuse idiopathic skeletal hyperostosis (DISH), also known as Forestier disease, is a noninflammatory condition which may be related to underlying metabolic disease. It is characterised by bone formation in the spine and entheses with prevalence rising with age.^1,2^

Although DISH is asymptomatic in many individuals, the presence of spinal ossifications can lead to spinal pain, stiffness and loss of motion. If upper cervical involvement occurs additional symptoms such as dysphagia may be observed.^2,3^

Mild cervical pain and dysphagia associated with DISH are typically managed with conservative treatment, including dietary measures, physical and swallowing therapy, nonsteroidal anti-inflammatory drugs, and muscle relaxants. In refractory cases, surgical intervention may be necessary for osteophyte removal, yielding positive outcomes.^2,4^

## CASE DESCRIPTION

A 74-year-old male, with already known metabolic syndrome - dyslipidaemia, type II diabetes and obesity (IMC 32 kg/m^2^) – presented for chronic mechanical cervicalgia, with progressive decrease of flexion, extension and axial rotation, and episodes of inflammatory pain treated with non-steroidal anti-inflammatory drugs as needed.

**Figure 1.**
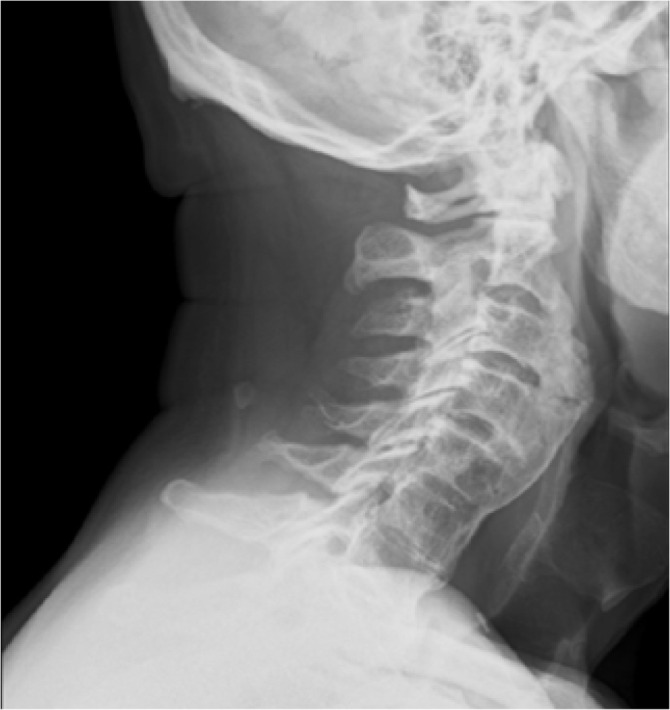
Lateral cervical spine x-ray showing anterior bone formation consistent with DISH with mass effect at the laryngopharynx.

He further mentioned a recent onset of odynophagia and oesophageal dysphagia for solids. Besides the decrease in axial mobility, the physical examination did not show any other relevant changes, such as gag reflex, dysphonia (wet voice), halitosis or bolus impaction. Latest endoscopic studies didn’t reveal any structural cause for the dysphagia. A cervical X-ray was required which showed a finding compatible with DISH with exuberant anterior vertebral body osteoproliferation (**Figure 1**).

**Figure 2.**
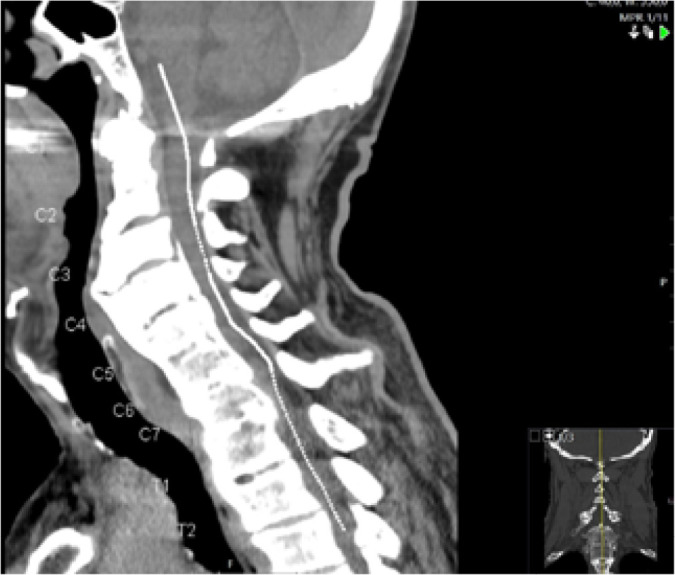
A sagittal Computed Tomographic (CT) scanning cervical spine showing anterior ossification particularly prominent at the level of C3-C4 with indentation on the posterior pharyngeal wall.

**Figure 3.**
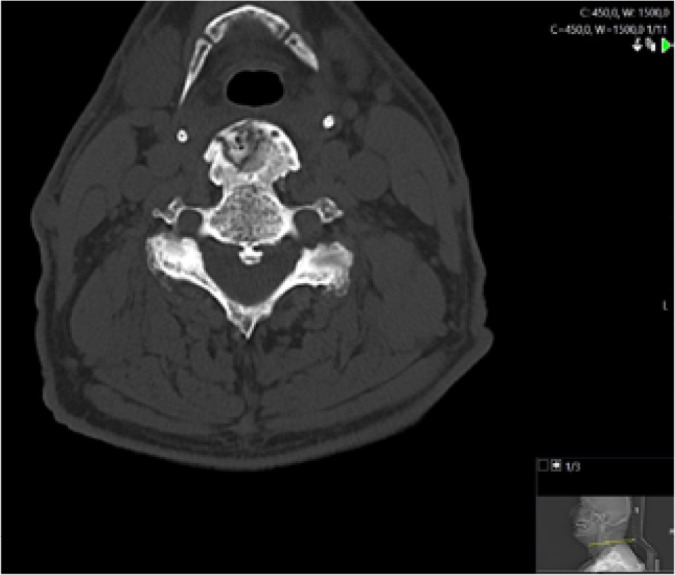
Axial CT scan at the C3-C4 level reveals prominent anterior ossification in close proximity to the laryngopharynx. At this level, calcification of the posterior longitudinal ligament is also observed.

Computed tomography (CT) scans of cervical spine can be seen in **Figures 2** and **3**.
